# The surgical treatment of Morgagni hernias in adults: a systematic review for the standardization of laparoscopic surgical repair

**DOI:** 10.1007/s13304-023-01677-3

**Published:** 2023-11-04

**Authors:** Elena Schembari, Elisa Reitano, Maria Sofia, Saverio Latteri, Gaetano La Greca

**Affiliations:** 1 University of Catania, Catania, Italy; 2 IRCAD Research Institute Against Digestive Cancer, Strasbourg, France; 3 General Surgery, Cannizzaro Hospital, Catania, Italy; 4 Department of Surgical Sciences and Advanced Technologies “G.F. Ingrassia”, Cannizzaro Hospital, University of Catania, Catania, Italy

**Keywords:** Morgagni hernia, Laparoscopic repair, Adults

## Abstract

A Morgagni hernia is a congenital diaphragmatic hernia that is rarely diagnosed in adults, and the technique for its repair has not yet been standardized. This review will give an overview of the different laparoscopic methods reported by other authors, highlighting the key points indicating a good repair to help standardize the technique. A systematic review of the available articles on PubMed was conducted according to PRISMA 2020 by two authors independently in May 2022. Only articles written in English were included. A total of 180 case reports of laparoscopic Morgagni’s hernia repair procedures were found; direct repair was performed in 59 patients, mesh was used in 119 patients, and mesh was not used in 2 patients. The hernia sac was removed in 71 patients, and the defect was closed before mesh placement in 49 patients. Nonabsorbable, dual or biologic mesh was used. The mean operative time was 92.65 min for direct repair and 84.11 min for mesh repair. One recurrence was reported in the direct repair series. The optimal method of repair has not yet been identified. The laparoscopic approach is associated to fewer complications and facilitates a faster recovery than the open approach. Several manoeuvres have been reported to help surgeons, who are not trained in laparoscopic knotting, perform extracorporeal knotting. Mesh should be placed when tension is too high after a direct repair or when primary closure cannot be achieved.

**Supplementary Information:**

The online version contains supplementary material available at 10.1007/s13304-023-01677-3.

## Introduction

The Morgagni hernia (MH) was described as a diaphragmatic defect behind the sternum by the Italian anatomist Morgagni in 1769 [1]. It accounts for 3% of all diaphragmatic hernias, and because it is congenital in nature, it is more frequently found in children than in adults. However, cases of MH in adults have been reported in the literature. The diagnosis can be incidental, such as during investigations or surgical procedures performed for other reasons or in an emergency setting due to incarceration [2]. Different approaches have been proposed for MH repair, including thoracotomy, thoracoscopy, laparotomy, and laparoscopy. The laparoscopic approach seems to have a similar complication rate to the other approaches but offers a better view of the diaphragm and quicker recovery [3]. However, the technique has not yet been standardized. In our review, we give an overview of the different laparoscopic methods reported by other authors. Our main aims were to highlight the key points for a good repair to try to standardize the technique.

## Method

Two authors independently and systematically reviewed articles available in PubMed in May 2022. This work was reported in line with PRISMA 2020 (Preferred Reporting Items for Systematic Reviews and Meta-Analyses) guidelines. The following search terms were used: “Morgagni hernia” AND “adult” AND “repair”. All types of studies written in English and case reports of laparoscopic Morgagni hernia repair in adults were included. Descriptive statistics were used to characterize the study population.

## Results

**Fig. 1:**
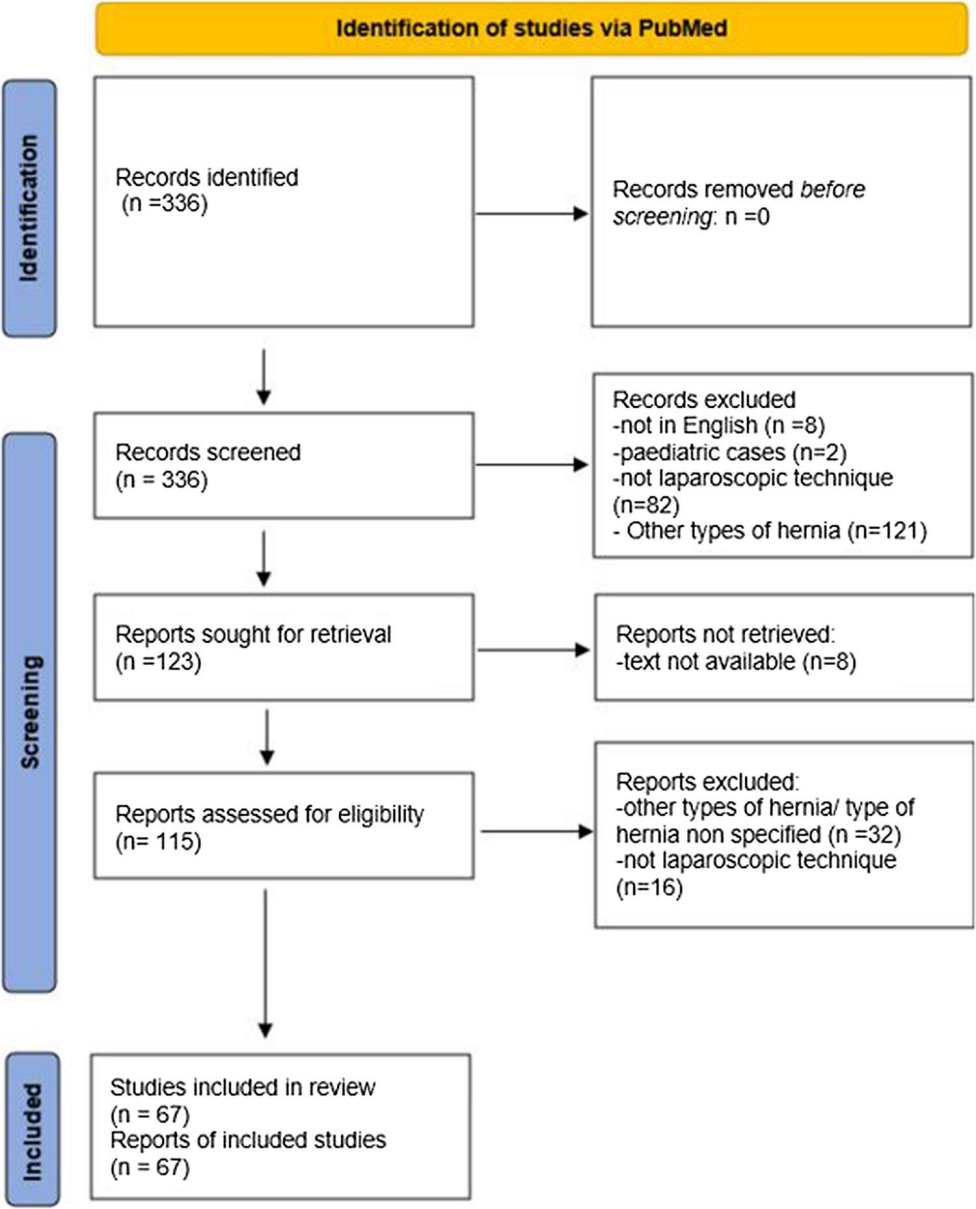
Prisma flow diagram

For this analysis, 67 publications were considered relevant (Fig. 1). A total of 180 case reports of laparoscopic Morgagni’s hernia repair were found, including four procedures where a SILS (single incision laparoscopic surgery) port was used [4, 5]. Direct repair was performed in fifty-nine patients, a mesh was used in 119 patients, and the technique was not reported (NR) in 2 cases. Eleven repairs were emergency procedures, two patients had Down’s syndrome, and one of them was a recurrence.

The hernia sac was removed in 71 patients, not excised in 48 patients, partly excised in one patient and was not reported in the remaining cases (Table 1).

**Table 1:** Laparoscopic Morgagni hernia repair: intraoperative data

Total cases (*n* = 180) Emergency (*n* = 11) elective (*n* = 169)
Emergency setting Direct repair (1) Mesh repair (10)
Hernia sac (*n*) Excised (71) Not excised (48) Partly excised (1)
Type of repair (*n*) Direct (59) Mesh (119) Not reported (2)
Defect suturing (*n*) Direct (59) Mesh (49)
Type of used mesh (*n*) Non-absorbable (34) Dual mesh (49) Biologic mesh (3) Not reported (32)
Mean operative time (minutes) Direct repair (92.65) Mesh repair (84.11)

Some authors preferred a mesh hernia repair with or without suturing the defect. The defect was closed before mesh placement in 49 cases. The size of the mesh had to be sufficient to overlap the defect by 1.5–2 cm [6]. Different types of mesh were used, and similarly, different methods were adopted to fix them, such as stitches, protacks, absorbable tacks, fibrin glue, and cyanoacrylate drops [7].

Nonabsorbable mesh (prolene, polypropylene, PTFE) was used in 34 patients, dual mesh (e.g. Parietex composite, Proceed, Bard composite, Dynamesh, Ventralight, Physiomesh) was used in 49 cases, biologic in 3 cases, bioabsorbable (Phasix) in one case, and the type of mesh was not reported in the remaining 32 cases. Ten of the eleven emergency patients were treated with mesh repair [nine with synthetic mesh, one not reported [2]].

The mean operative time was reported in 22 studies (31 direct repair procedures and 53 mesh repair procedures), and the operative time was 92.65 min for direct repair and 84.11 min for mesh repair.

The postoperative complications (Table 2) observed in the direct repair group were one case of sputum retention that was treated with chest physiotherapy [8], pulmonary oedema [9], hypoxia [10], and seroma that required drainage [11]. In the mesh repair group, there was a lower respiratory tract infection [12], a pneumothorax [2], three haematomas (one radiologically drained (5, 13) and one surgically treated [14]), two seromas that were treated conservatively [15, 16] and one that required drainage [5], a case of mild atelectasis of the lungs [17], a port site hernia [13], a port site seroma [5], one case of pleural effusion [18], and a haemothorax that was drained [18].

**Table 2:** Laparoscopic Morgagni hernia repair: length of hospital stay, postoperative complications, follow-up and recurrence rate

Complications Direct repair: Sputum retention (1) Pulmonary oedema (1) Hypoxia (1) Seroma (1) (drained) Mesh repair Low respiratory tract infection (1) Pneumothorax (1) Haematoma (3) (1 radiologically drained, 1 surgically drained) Seroma (3) (1 drained) Mild atelectasis (1) Port site hernia (1) Port site seroma (1) Pleural effusions (1) Haemothorax (1) (drained) Technique not specified Sinus infection (1) (oral antibiotics) Hypoxemia (2) Pneumonia (1) Acute kidney injury (1) Pleural effusion (1)
Mean hospital stay (days) Direct repair (2.6) Mesh repair (7.6)
Recurrence rate (*n*) Direct repair (1) Mesh repair (0)

Other authors reported the following complications without specifying if they happened in the direct or mesh repair group: a sinus infection treated with oral antibiotics [19], two cases of hypoxemia [20], a case of pneumonia [20], an acute kidney injury (AKI) [20], and a case of pleural effusion [20]. No cases of mesh infection were reported.

The mean hospital stay was 2.6 days after direct repair (57 patients) versus 7.6 days after mesh repair (89 patients). One of the patients who underwent mesh repair remained in the hospital for 26 days because they were waiting for a rehabilitation bed due to a recent orthopaedic surgery.

The follow-up period varied considerably, ranging from 1 to 120 months. Only one recurrence [11] was reported in the direct repair series, which occurred six months after the surgery and was treated with an open mesh repair. No recurrences have been reported in the mesh series.

## Discussion

A MH is rare, especially in adulthood [21], and there are no clear guidelines for its repair. Compared to open techniques, the laparoscopic approach appears to be safe, due to its low morbidity and short hospital stay [3]. However, there is no standardized technique.

In 1996, Orital et al. suggested [22] that the laparoscopic technique allowed a better view of the diaphragm compared to the open approach. They described the “abdominal wall lifting technique”, which involves the use of two Kirschner wires to lift the abdominal wall; thus, the hernia can be treated using laparoscopic tools without creating pneumoperitoneum, but no other articles have been published about this procedure.

Whether the hernia sac should be removed remains unclear because leaving it could increase the risk of postoperative seroma formation, while its resection could lead to circulatory and respiratory complications due to damage to the mediastinal structures. According to our review, the hernia sac was not removed in the four patients who developed a seroma, while haemothorax occurred in a patient who had the hernia sac removed. Aiming to resolve this dilemma, Ben-Yacoov et al. [23] proposed extrasaccular dissection, avoiding the excision of the medial part of the sac, which is the riskiest phase. Ikarashi et al. [16] concluded their article stating that sac excision should be performed according to the density of the adhesions and the patient’s condition. Edye et al. [24] stated that sac removal reduces the recurrence rate of paraoesophageal hernias, and, in our review, we reported recurrence in a patient who underwent direct repair without sac excision. However, because of the lack of data, it is hard to say if there is a correlation between the recurrence rate and hernia sac removal.

Regarding direct repair, apart from traditional laparoscopic stitches, other techniques have been introduced to facilitate repair. According to the technique proposed by La Greca et al. [25] and later also reported by others [26], nonabsorbable stitches were placed through the abdominal wall and the free diaphragmatic border, and both ends of each suture were brought outside using a Reverdin needle holder. Before tying the knots, all the sutures were pulled together to ensure good closure of the defect and to reduce tension during knotting. Costa Almeida et al. [27] and Park et al. [28] applied nonabsorbable sutures with straight needles passed through the abdominal wall and the diaphragm, creating a U shape with pledgets. Then, a spinal cannula was introduced to facilitate the retrieval of the straight needle. The sutures were then tied extracorporeally.

There is also no clear consensus about the indications for direct versus mesh repair. All authors did not report the size of the defect, so it is difficult to establish the threshold for mesh placement. Ben-Yaacov et al. [23] advised mesh repair when the area of the defect was over 20–30 cm^2^. It should also be considered that some authors reported the area rather than the diameter of the hernia, and it is well known that the shape of the defect is usually oval rather than circular with a transverse diameter that is longer than the anteroposterior diameter [29]. Moreover, the size of the defect is usually smaller when measured on CT than intraoperatively because of the pneumoperitoneum [15]. We believe that this overestimation of the size of the defect could lead to unnecessary use of mesh for hernias that could be repaired directly. Zaharie et al. [30] advised complete desufflation of the abdomen during knotting and reinsufflation afterwards. In our opinion, gentle pressure on the anterior abdominal wall should be applied to assess whether reapproximating the anterior and posterior edges of the defect is possible. In addition, once the sutures have been placed, reducing the pressure of insufflation would allow the surgeon to see if the closure of the defect is under tension; if so, mesh should be used to reinforce the repair [19]. On one hand, mesh placement could theoretically reduce the chance of recurrence even if there is no evidence to support this thus far; on the other hand, it surely increases the costs of surgery. Kumar et al. [31] had to use a polypropylene mesh in one of their three cases instead of the more expensive composite mesh due to the cost factor. Overall, different types of mesh have been used, varying from biological to dual mesh and polypropylene. To avoid adhesion formation between the mesh and the intra-abdominal organs, covering the mesh with peritoneum [32], omental fat or flaps of falciform ligament has been suggested [33]. Another disadvantage of mesh repair is related to tack placement. In fact, the diaphragm is extremely thin, especially medially, where important structures are present [23], and could be damaged by tacks, leading to life-threatening consequences.

It is interesting to note that ten of the eleven emergency patients were treated with mesh repair, and none of them developed mesh infection.

There are a few limitations to this study. The first is that the size of the defect was not reported in all the articles. Therefore, it is quite difficult to compare the outcomes of direct repair vs. mesh repair. The second limitation is that all the studies are small case series, so the lack of large populations or randomized studies does not allow us to obtain statistically significant data. In addition, the follow-up period varied considerably, ranging from 1 month to 10 years, which could affect the reported recurrence rate.

## Conclusion

The optimal method of repair has not been identified because of the rarity of this condition and the lack of randomized trials. The laparoscopic approach has been considered a good option [34] because it allows a better view of the diaphragm [33], causes fewer complications, and facilitates a faster recovery than the open approach [11]. Extracorporeal knotting can be easily performed by any surgeon even if they are not very well trained in laparoscopic knotting, and several manoeuvres have been reported to facilitate it. It does not appear that the hernia sac must excised to achieve good outcomes. Mesh should be placed when the tension is too high after direct repair or when primary closure cannot be achieved.

### Supplementary Information

Below is the link to the electronic supplementary material.

Supplementary file1 (DOCX 55 KB)

Supplementary file2 (DOCX 46 KB)

## Data Availability

Data are available if requested.
