# Supplementary material for: The surgical treatment of Morgagni hernias in adults: a systematic review for the standardization of laparoscopic surgical repair
Source: Updates Surg. 2023 Nov 4;76(3):839–44. doi: 10.1007/s13304-023-01677-3 (PMC11130068; doi:10.1007/s13304-023-01677-3)
Supplement: Supplementary file 1 — Supplementary file1 (DOCX 55 KB) [file 13304_2023_1677_MOESM1_ESM.docx]

| **Author** | **Year** | **Setting** | **Laparoscopic repair** | **Direct repair** | **Defect closure** | **Type of suture** | **Mesh** | **Type of mesh** | **Hernia sac excised** | **Operation lenght (minutes)** |
| --- | --- | --- | --- | --- | --- | --- | --- | --- | --- | --- |
| Park A et al. (1) | 2014 | Elective | 13 | 13 |  | non-absorbable transabdominal stitches on a tapered Keith needle and 18-gauge spinal cannulla | 0 |  | NR | NR |
| Ortenzi M et al. (2) | 2020 | Elective | 9 | 9 |  | running self-fixating suture (V-loc) | 0 |  | NR | 80±25 |
| Ağalar C et al. (3) | 2019 | Elective | 3 | 3 |  | interrupted sutures of prolene 1 | 0 |  | No | NR |
| Misra RP et al. (4) | 2011 | Elective | 2 | 2 |  | surgical sutures passed transfacially with a laparoscopic suture passer | 0 |  | 2 | NR |
| Contini S et al. (5) | 1999 | Emergency | 1 | 1 |  | interrupted 3-0 prolene stitches with extracorporeal knotting | 0 |  | no | 45 |
| Angrisani L et al. (6) | 2000 | Elective | 1 | 1 |  | interrupted polyester stitches (Ethibond-Ethicon) using the external knot-tying technique. | 0 |  | NR | NR |
| La Greca G et al. (7) | 2001 | Elective | 1 | 1 |  | sutures introduced with a Reverdin needle | 0 |  | No | 12 |
| Lee KF et al. (8) | 2002 | Elective | 1 | 1 |  | 2-0 polypropylene on a long straight needle through a 3-mm skin stab wound to approximate the rectus sheath and the anterior diaphragm under laparoscopic assistance | 0 |  | No | NR |
| Richardson WS et al. (9) | 2002 | Elective | 1 | 1 |  | Neurolon 0 | 0 |  | NR | 140 |
| Cokmez A et al. (10) | 2003 | Elective | 1 | 1 |  | intracorporeal sutures and knotting | 0 |  | NR | 115 |
| De Paolis P et al. (11) | 2007 | Elective | 1 | 1 |  | silk and polypropylene sutures | 0 |  | NR | NR |
| Yamaguchi S et al. (12) | 2012 | Elective | 1 | 1 |  | interrupted sutures | 0 |  | no | NR |
| Tone K et al. (13) | 2013 | Elective | 1 | 1 |  | NR | 0 |  | NR | NR |
| Costa Almeida C et al. (14) | 2019 | Elective | 1 | 1 |  | intermittent extra-abdominal transfascial non-absorbable sutures with straight needle | 0 |  | no | NR |
| Kuikel S et al. (15) | 2021 | Elective | 1 | 1 |  | non-absorbable interrupted stitches | 0 |  | no | NR |
| Zaharie F et al. (16) | 2022 | Elective | 4 | 4 |  | non-reabsorbable monofilament sutures using a Reverdin needle | 0 |  | 4 | 110 |
| Rau HG et al. (17) | 1994 | Elective | 1 | 0 | no |  | 1 | Marlex mesh stapled to the margins | 1 | NR |
| Orita M et al. (18) | 1997 | Elective | 1 | 0 | 1 | 2-0 silk interrupted sutures with intra-abdominal suturing technique | 1 | Prolene mesh fixed with hernia stapler | 1 | NR |
| Nguyen T et al. (19) | 1998 | Elective | 1 | 0 | no |  | 1 | Marlex fixed with 2-0 prolene sutures | 1 | NR |
| Filipi CJ et al. (20) | 2000 | Elective | 1 | 0 | no |  | 1 | Goretex | NR | NR |
| White DC et al. (21) | 2002 | Elective | 1 | 0 | NR |  | 1 | polytetrafluoroethylene mesh fixed with stitches and stapler | no | NR |
| Marín-Blazquez AA et al. (22) | 2004 | Elective | 1 | 0 | no |  | 1 | Parietex composite mesh fixed with tacks | NR | NR |
| Tarim A et al. (23) | 2004 | Elective | 1 | 0 | 1 |  | 1 | polypropilene mesh stapled to the edges | NR | NR |
| Sherigar JM et al. (24) | 2005 | Elective | 1 | 0 | no |  | 1 | polypropylene mesh fixed with prolene stiches | no | 120 |
| Kelly MD (25) | 2007 | Emergency | 1 | 0 | NR |  | 1 | polypropylene mesh fixed with Protack | 1 | NR |
| Pallati PK et al. (26) | 2008 | Elective | 1 | 0 | no |  | 1 | biologic mesh fixed with tacks and sutures | NR | NR |
| Kim HR et al. (27) | 2009 | Elective | 1 | 0 | no |  | 1 | Proceed mesh | 1 | NR |
| Khandelwal S et al. (28) | 2011 | Elective | 1 | 0 | no |  | 1 | mesh fixed with transfascial stitches anteriorly, with matress sutures and AbsorbaTacks on the other edges | 1 | NR |
| Rattay T et al. (29) | 2011 | Elective | 1 | 0 | no |  | 1 | Composite mesh | NR | NR |
| Chiou G et al. (30) | 2012 | Elective | 1 | 0 | no |  | 1 | Parietex fixed with interrupted sutures | NR | NR |
| Chick JF et al. (31) | 2012 | Emergency | 1 | 0 | NR |  | 1 | NR | NR | NR |
| Vassileva CM et al. (32) | 2012 | Elective | 1 | 0 | no |  | 1 | mesh fixed with interrupted non-reabsorbable sutures | 1 | NR |
| Stone ML et al. (33) | 2012 | Emergency | 1 | 0 | no |  | 1 | prolene mesh fixed with polyester sutures | 1 | NR |
| Chen Y et al. (34) | 2013 | Emergency | 1 | 0 | no |  | 1 | dual layer polytetrafluoroethylene-polypropylene mesh fixed with interrupted sutures and AbsorbaTacks | NR | NR |
| Kaida T et al. (35) | 2014 | Elective | 1 | 0 | no |  | 1 | Parietex composite fixed to the anterior abdo wall with a hernia stapler and posteriorly with non-reabsorbable stitches. The double-crown technique was adopted to fix the mesh around the hernia orifice | no | 139 |
| Kashiwagi H et al. (36) | 2014 | Elective | 1 | 0 | no |  | 1 | Goretex dual mesh | partial | 157 |
| Jakhmola CK et al. (37) | 2015 | elective | 1 | 0 | NR |  | 1 | dual mesh secured with tackers and intracorporeal silk stitches close to the pericardium | no | NR |
| Ikarashi M et al. (38) | 2015 | elective | 1 | 0 | no |  | 1 | bard composix mesh fixed with AbsorbaTack™ and 2 transabdominal suture at the anterior abdo wall | no | 53 |
| Li S et al. (39) | 2015 | Elective | 1 | 0 | no |  | 1 | pericardium patch fixed with polypropylene stitches | no | NR |
| Chamary SL et al. (40) | 2015 | Elective | 1 | 0 | no |  | 1 | Proceed | NR | NR |
| Sahsamanis G et al. (41) | 2017 | Elective | 1 | 0 | 1 | NR | 1 | non-absorbable mesh | 1 | NR |
| Kim DK et al. (42) | 2017 | Elective | 1 | 0 | no |  | 1 | dualmesh fixed with transfascial stitches | NR | NR |
| Badic B et al. (43) | 2017 | Elective | 1 | 0 | 1 | non-absorbable matress suture | 1 | synthetic mesh | 1 | NR |
| Liu S et al. (44) | 2018 | Elective | 1 | 0 | NR |  | 1 | Pari-etexTMcomposite fixed with 1.0 Prolene | NR | NR |
| Hoyuela C et al. (45) | 2019 | Elective | 1 | 0 | 1 | transparietal interrupted 2/0 polyester sutures | 1 | Dynamesh IPOM® fixed with a centra stiche and then with cyanoacrylate drops | 1 | 85 |
| Azar N et al. (46) | 2019 | Elective | 1 | 0 | no |  | 1 | A sleeve of Parietex composite mesh cut to act as a suture bolster+ Parietex fixed with absorbable tacks, V-loc suture and fibrin glue | 1 | NR |
| Ben-Yaacov a et al. (47) | 2020 | Elective | 1 | 0 | no |  | 1 | Parietex fixed with AbsorbaTack™ Covidien | no | 150 |
| Mohamed M et al. (48) | 2020 | Elective | 1 | 0 | 1 | NR | 1 | NR | NR | NR |
| Târcoveanu E et al. (49) | 2018 | Elective | 4 | 2 | 2 | non-resorbable | 2 | NR | 4 | 65 |
| Minneci PC et al. (50) | 2004 | Elective | 2 | 0 | no |  | 2 | polypropilene mesh fixed with ProTack | NR | NR |
| Altinkaya N et al. (51) | 2009 | Elective emergency | 2 | 0 | no |  | 2 | polypropilene mesh fixed with ProTack | no | 82 ± 31 |
| Godazandeh G et al. (52) | 2012 | Elective | 2 | 0 | no |  | 2 | dual-sided mesh fixed with Protack | 2 | NR |
| Shakya VC (53) | 2015 | elective | 2 | 0 | 2 | polypropilene suture | 2 | polypropylene mesh fixed with prolene stitches | no | NR |
| Soldo I et al. (54) | 2017 | Elective | 3 | 0 | no |  | 3 | Proceed (n=2),Composite(n=1) | no | NR |
| Ryan JM et al. (55) | 2018 | Elective | 3 | 0 | 3 | 2-0 propylene sutures | 3 | Parietex™ polyester composite mesh fixed with AbsorbaTack™ | 3 | NR |
| Kumar A et al. (56) | 2020 | Elective | 3 | 0 | 3 | polypropylene suture | 3 | Composite (n=2), polypropilene (n=1) | NR | 125.7 |
| Durak E et al. (57) | 2007 | Elective | 5 | 1 | 1 |  | 4 | Parietex mesh fixed with sutures and tacks | no | 95 |
| Leeflang E et al. (58) | 2021 | Elective | 12 | 7 | 12 | permanent interruptes sutures delivered through the abdominal wall with a suture passing device | 5 | Composite mesh fixed with permanent interrupted sutures | 12 | 93 |
| Yavuz N et al. (59) | 2006 | Elective | 5 | 0 | no |  | 5 | ePTFE (n=4) and bovine pericardium (n=1) fixed to the anterior abdominal wall with prolene sutures drawn out with endoclose and the remaining part fixed with Protacks | no | 69 |
| Young MC et al. (60) | 2018 | Elective | 10 | 3 | 3 | NR | 7 | NR | NR | 138 |
| Oppelt PU et al. (61) | 2020 | Elective | 7 | 0 | 7 | non-resorbable, interrupted sutures | 7 | NR - doesn't specific open/lap: coated (n=6)/ uncoated(n=4)/ Gore-Tex (n=1) ,mesh fixed with single suture or endostaplers | 7 | 120 |
| Arikan S et al. (62) | 2018 | Elective | 12 | 0 | 4 |  | 12 | polypropylene (n=3), dual mesh (n=1) | 8 | NR |
| Kao AM et al. (63) | 2021 | Elective (n=13) emergency (n=2) | 15 | 4 | 4 | permanent suture through transfascial sutures with a suturepasser or intracorporeal suturing. | 11 | PTFE and coated polypropylene fixed with trasfacial stitches, running or interrupted stitches and tacks | 15 | NR |
| Onafowokan OO et al. (64) | 2021 | Elective | 1 | 0 | 1 | multiple transfascial sutures | 1 | bioabsorbable Phasix™ ST Mesh | 1 | NR |
| Pazouki A et al. (65) | 2022 | Emergency | 1 | 0 | 1 | prolene sutures | 1 | dual mesh | 1 | 120 |
| Altın Ö et al. (66) | 2021 | Elective (n=13) Emergency (n=2) | 15 | 0 | 15 | polpypropilene stitches | 15 | Ventralight (n=8), Physiomesh (n=7) | no | 69.8 |
| Taskin M et al. (67) | 2002 | Elective | 2 | NR | NR |  | NR |  | NR | NR |

Table 1 supplement: Laparoscopic Morgagni hernia repair: intra-operative data

# References

1. **Park A, Doyle C. Laparoscopic Morgagni hernia repair: how I do it. J Gastrointest Surg. 2014 Oct;18(10):1858-62. doi: 10.1007/s11605-014-2552-y. Epub 2014 Jun 5. PMID: 24898515.**

**2. *Ortenzi M, Balla A, Paganini AM, Biondini G, Lezoche G, Ghiselli R, Guerrieri M. Laparoscopic repair of giant Morgagni hernia by direct suturing with V-Loc. Minerva Chir. 2020 Oct;75(5):298-304. doi: 10.23736/S0026-4733.20.08477-1. PMID: 33210525.***

**3. Ağalar C, Atila K, Arslan NÇ, Derici ZS, Bora S. Adult morgagni hernia: a single center experience of 5 cases and review of literature. Turk J Surg. 2019 Dec 16;35(4):321-324. doi: 10.5578/turkjsurg.3929. PMID: 32551430; PMCID: PMC7282460.**

**4. Misra RP, Schwartz JD. A simplified technique of full-thickness transabdominal laparoscopic repair of Morgagni hernia. J Thorac Cardiovasc Surg. 2011 Feb;141(2):594-5. doi: 10.1016/j.jtcvs.2010.06.046. Epub 2010 Aug 9. PMID: 20692000.**

**5. *Contini S, Dalla Valle R, Bonati L, Zinicola R. Laparoscopic repair of a Morgagni hernia: report of a case and review of the literature. J Laparoendosc Adv Surg Tech A. 1999 Feb;9(1):93-9. doi: 10.1089/lap.1999.9.93. PMID: 10194700.***

**6. *Angrisani L, Lorenzo M, Santoro T, Sodano A, Tesauro B. Hernia of foramen of Morgagni in adult: case report of laparoscopic repair. JSLS. 2000 Apr-Jun;4(2):177-81. PMID: 10917128; PMCID: PMC3015372.***

**7. La Greca G, Fisichella P, Greco L, Stefano A, Russello D, Latteri F. A new simple laparoscopic-extracorporeal technique for the repair of a Morgagni diaphragmatic hernia. Surg Endosc. 2001 Jan;15(1):99. doi: 10.1007/s004640040027. PMID: 11285540.**

**8. Lee KF, Chung DP, Leong HT. Laparoscopic repair of morgagni's hernia with percutaneous placement of suture. J Laparoendosc Adv Surg Tech A. 2002 Feb;12(1):65-8. doi: 10.1089/109264202753486966. PMID: 11908486.**

**9. *Richardson WS, Bolton JS. Laparoscopic repair of congenital diaphragmatic hernias. J Laparoendosc Adv Surg Tech A. 2002 Aug;12(4):277-80. doi: 10.1089/109264202760268078. PMID: 12269497.***

**10. *Cokmez A, Durak E. Laparoscopic repair of Morgagni hernia and paraesophageal hernia on the same patient. Surg Endosc. 2003 Apr;17(4):660. doi: 10.1007/s00464-002-4238-x. Epub 2003 Feb 10. PMID: 12574935.***

**11. De Paolis P, Mazza L, Maglione V, Fronda GR. Laparoscopic repair of Morgagni hernia and cholecystectomy in a 40-year-old male with Down's sindrome. Report of a case. Minerva Chir. 2007 Jun;62(3):197-200. PMID: 17519846.**

**12. *Yamaguchi S, Marshall MB. Outpatient laparoscopic repair of a Morgagni hernia. Surg Innov. 2013 Dec;20(6):NP38-9. doi: 10.1177/1553350612444170. Epub 2012 Apr 18. PMID: 22517330.***

**13. *Tone K, Kiryu I, Yoshida M, Tsuboi K, Takagi M, Kuwano K. Morgagni hernia with respiratory failure aggravated by noninvasive positive pressure ventilation: a case report and overview of the literature. Respir Investig. 2014 May;52(3):203-8.* doi: 10.1016/j.resinv.2013.11.001. Epub 2014 Jan 18. PMID: 24853023.**

**14. Costa Almeida C, Caroço TV, Nogueira O, Infuli A. Laparoscopic repair of a Morgagni hernia with extra-abdominal transfascial sutures. BMJ Case Rep. 2019 Jan 29;12(1):e227600. doi: 10.1136/bcr-2018-227600. PMID: 30700463; PMCID: PMC6352826.**

**15. *Kuikel S, Shrestha S, Thapa S, Maharjan N, Kandel BP, Lakhey PJ. Morgagni hernia in adult: A case report. Int J Surg Case Rep. 2021 Aug 6;85:106286.* doi: 10.1016/j.ijscr.2021.106286. Epub ahead of print. PMID: 34388911; PMCID: PMC8358627.**

**16. *Zaharie F, Valean D, Popa C, Mois E, Graur F, Munteanu D, Schlanger D, Ciocan A, Puia C, Al Hajjar N. Surgical technique in the laparoscopic repair of Morgagni hernia in adults. How do we do it? Hernia. 2022 Jan 10.* doi: 10.1007/s10029-021-02559-6. Epub ahead of print. PMID: 35013791.**

**17. Rau HG, Schardey HM, Lange V. Laparoscopic repair of a Morgagni hernia. Surg Endosc. 1994 Dec;8(12):1439-42. doi: 10.1007/BF00187355. PMID: 7878515.**

**18. Orita M, Okino M, Yamashita K, Morita N, Esato K. Laparoscopic repair of a diaphragmatic hernia through the foramen of morgagni. Surg Endosc. 1997 Jun;11(6):668-70. doi: 10.1007/s004649900418. PMID: 9171131.**

**19. *Nguyen T, Eubanks PJ, Nguyen D, Klein SR. The laparoscopic approach for repair of Morgagni hernias. JSLS. 1998 Jan-Mar;2(1):85-8. PMID: 9876719; PMCID: PMC3015255.***

**20. *Filipi CJ, Marsh RE, Dickason TJ, Gardner GC. Laparoscopic repair of a Morgagni hernia. Surg Endosc. 2000 Oct;14(10):966-7. doi: 10.1007/s004649901207. Epub 2000 Aug 22. PMID: 11287983.***

**21. White DC, McMahon R, Wright T, Eubanks WS. Laparoscopic repair of a Morgagni hernia presenting with syncope in an 85-year-old woman: case report and update of the literature. J Laparoendosc Adv Surg Tech A. 2002 Jun;12(3):161-5. doi: 10.1089/10926420260188047. PMID: 12184900.**

**22. Marín-Blazquez AA, Candel MF, Parra PA, Méndez M, Ródenas J, Rojas MJ, Carrión F, Madrigal M. Morgagni hernia: repair with a mesh using laparoscopic surgery. Hernia. 2004 Feb;8(1):70-2. doi: 10.1007/s10029-003-0145-x. . Epub 2003 Nov 22. PMID: 14634839.**

**23. *Tarim A, Nursal TZ, Yildirim S, Ezer A, Caliskan K, Törer N. Laparoscopic repair of bilateral morgagni hernia. Surg Laparosc Endosc Percutan Tech. 2004 Apr;14(2):96-7. doi: 10.1097/00129689-200404000-00011. PMID: 15287610.***

**24. *Sherigar JM, Dalal AD, Patel JR. Laparoscopic repair of a Morgagni hernia. J Minim Access Surg. 2005 Jun;1(2):76-8. doi: 10.4103/0972-9941.16532. PMID: 21206651; PMCID: PMC3004110.***

**25. Kelly MD. Laparoscopic repair of strangulated Morgagni hernia. World J Emerg Surg. 2007;2:27. Published 2007 Oct 12. doi:10.1186/1749-7922-2-27.**

**26. Pallati PK, Puri V, Mittal SK. Gastric outlet obstruction secondary to Morgagni hernia: a case report. Hernia. 2008 Apr;12(2):209-12. doi: 10.1007/s10029-007-0287-3. Epub 2007 Oct 11. PMID: 17929108.**

**27. *Kim HR, Hong TH, Lee YS, et al. Elective laparoscopic repair after colonoscopic decompression for incarcerated morgagni hernia. Gut Liver. 2009;3(4):318-320. doi:10.5009/gnl.2009.3.4.318.***

**28. *Khandelwal S, Oelschlager BK. Video. Laparoscopic repair of congenital bilateral Morgagni hernia. Surg Endosc. 2011 Jun;25(6):2010. doi: 10.1007/s00464-010-1337-y. Epub 2011 Feb 7. PMID: 21298550.***

**29. *Rattay T, Ubhi S. Morgagni hernia--an uncommon cause of gastric outlet obstruction. BMJ Case Rep. 2011;2011:bcr0520114264. Published 2011 Aug 24. doi:10.1136/bcr.05.2011.4264.***

**30. *Chiou G, Tedesco M, Eisenberg D. Concurrent laparoscopic morgagni hernia repair and sleeve gastrectomy. J Laparoendosc Adv Surg Tech A. 2012 Dec;22(10):999-1002. doi: 10.1089/lap.2012.0293. Epub 2012 Oct 15. PMID: 23067069.***

**31. Chick JF, Chauhan NR, Lai JH, Khurana B. Incarcerated Morgagni hernia mimicking acute cholecystitis. Intern Emerg Med. 2012 Sep;7 Suppl 2:S169-71. doi: 10.1007/s11739-012-0818-5. Epub 2012 Jul 15. PMID: 22797947.**

**32. *Vassileva CM, Shabosky J, Boley T, Hazelrigg S. Morgagni hernia presenting as a right middle lobe compression. Ann Thorac Cardiovasc Surg. 2012;18(1):79-81. doi: 10.5761/atcs.cr.11.01690. Epub 2011 Sep 29. PMID: 21959196.***

**33. Stone ML, Julien MA, Dunnington GH Jr, Lau CL. Novel laparoscopic hernia of Morgagni repair technique. J Thorac Cardiovasc Surg. 2012 Mar;143(3):744-5. doi: 10.1016/j.jtcvs.2011.07.048. Epub 2011 Sep 15. PMID: 21924744; PMCID: PMC3288951.**

**34. Chen Y, Wykes J, Haveman JW, Apostolou C, Merrett ND. Laparoscopic repair of Morgagni hernia: an interesting case and complication. ANZ J Surg. 2013 Sep;83(9):688-9. doi: 10.1111/ans.12219. PMID: 23998468.**

**35. Kaida T, Ikeda A, Shimoda H, Sako H, Uchida H, Wada M, Ikeda K, Okusawa S, Watanabe M. Laparoscopic mesh repair of a Morgagni hernia using the double-crown technique: A case study. Asian J Endosc Surg. 2014 Nov;7(4):323-6. doi: 10.1111/ases.12120. . PMID: 25354379.**

**36. *Kashiwagi H, Kumagai K, Nozue M, Terada Y. Morgagni hernia treated by reduced port surgery. Int J Surg Case Rep. 2014;5(12):1222-4. doi: 10.1016/j.ijscr.2014.11.047. Epub 2014 Nov 20. PMID: 25437681; PMCID: PMC4275975.***

**37. *Jakhmola CK, Kumar V. Laparoscopic mesh repair of Morgagni hernia in an octogenarian patient. Med J Armed Forces India. 2015 Jan;71(1):79-81. doi: 10.1016/j.mjafi.2012.06.017. Epub 2012 Sep 28. PMID: 25609870; PMCID: PMC4297838.***

**38. Ikarashi M, Matsuda M, Murayama I, Fujii M, Takayama T. Laparoscopic repair of Morgagni hernia with composite mesh in an elderly woman: Report of a case. Asian J Endosc Surg. 2015 May;8(2):216-8. doi: 10.1111/ases.12161. PMID: 25913591.**

**39. *Li S, Liu X, Shen Y, Wang H, Feng M, Tan L. Laparoscopic repair of Morgagni hernia by artificial pericardium patch in an adult obese patient. J Thorac Dis. 2015 Apr;7(4):754-7.* doi: 10.3978/j.issn.2072-1439.2015.03.15. PMID: 25973243; PMCID: PMC4419301.**

**40. Chamary SL, Chamary VL. Laparoscopic features and repair of a combined left Spigelian hernia and left Morgagni diaphragmatic hernia. Ann R Coll Surg Engl. 2015 Mar;97(2):e25-6. doi: 10.1308/003588414X14055925060596. PMID: 25723678; PMCID: PMC4473424.**

**41. *Sahsamanis G, Terzoglou A, Theodoridis C, Kiakou M, Mitsopoulos G, Deverakis T, Dimitrakopoulos G. Laparoscopic repair of an excessive Morgagni hernia in an adult presenting as upside-down stomach. Int J Surg Case Rep.* 2017 Nov 11;41:443-445. doi: 10.1016/j.ijscr.2017.11.014. PMID: 29546012; PMCID: PMC5702857.**

**42. *Kim DK, Moon HS, Jung HY, Sung JK, Gang SH, Kim MH. An Incidental Discovery of Morgagni Hernia in an Elderly Patient Presented with Chronic Dyspepsia. Korean J Gastroenterol. 2017 Jan 25;69(1):68-73. doi: 10.4166/kjg.2017.69.1.68. PMID: 28135794.***

**43. *Badic B, Bail JP. Laparoscopic repair of Morgagni hernia - a video vignette. Colorectal Dis. 2017 Mar;19(3):302. doi: 10.1111/codi.13615. PMID: 28251785.***

**44. *Liu S, Aydinli HH, Meytes V. Laparoscopic repair of a massive Morgagni hernia - a video vignette. Colorectal Dis. 2018 Nov;20(11):1054. doi: 10.1111/codi.14420. Epub 2018 Oct 3. PMID: 30216636.***

**45. Hoyuela C, Juvany M, Guillaumes S. Cyanoacrylate for Safer Mesh Fixation During Laparoscopic Repair of Morgagni Hernia. Ann Thorac Surg. 2020 Apr;109(4):e305-e307. doi: 10.1016/j.athoracsur.2019.10.019. Epub 2019 Nov 27. PMID: 31785292.**

**46. *Azar N, Azar R, Robertson K, Gupta P. Innovative use of mesh bolster for adult Morgagni hernia repair. J Surg Case Rep. 2019 Jul 3;2019(7):rjz205. doi: 10.1093/jscr/rjz205. PMID: 31289635; PMCID: PMC6607960.***

**47. Ben-Yaacov A, Menasherov N, Bard V. Repair of a recurrent symptomatic hernia through the foramen of Morgagni: a case study and review of the literature. J Surg Case Rep. 2020 Jul 31;2020(7):rjaa230. doi: 10.1093/jscr/rjaa230. . PMID: 32760492; PMCID: PMC7394129.**

**48. *Mohamed M, Al-Hillan A, Shah J, Zurkovsky E, Asif A, Hossain M. Symptomatic congenital Morgagni hernia presenting as a chest pain: a case report. J Med Case Rep. 2020 Jan 18;14(1):13. doi: 10.1186/s13256-019-2336-9. .* PMID: 31952551; PMCID: PMC6969475.**

**49. *Târcoveanu E, Georgescu Ș, Vasilescu A, Andronic D, Dănilă N, Lupaşcu C, Bradea C. Laparoscopic Management in Morgagni Hernia - Short Series and Review of Literature. Chirurgia (Bucur). 2018 Jul-Aug;113(4):551-557.* doi: 10.21614/chirurgia.113.4.551. PMID: 30183586.**

**50. *Minneci PC, Deans KJ, Kim P, Mathisen DJ. Foramen of Morgagni hernia: changes in diagnosis and treatment. Ann Thorac Surg. 2004 Jun;77(6):1956-9. doi: 10.1016/j.athoracsur.2003.12.028. PMID: 15172245.***

**51. *Altinkaya N, Parlakgümüş A, Koc Z, Ulusan S. Morgagni hernia: diagnosis with multidetector computed tomography and treatment. Hernia. 2010 Jun;14(3):277-81. doi: 10.1007/s10029-009-0608-9. Epub 2009 Dec 24. PMID: 20033748.***

**52. Godazandeh G, Mortazian M. Laparoscopic Repair of Morgagni Hernia Using Polyvinylidene Fluoride (PVDF) Mesh. Middle East J Dig Dis. 2012 Oct;4(4):232-5. PMID: 24829663; PMCID: PMC3990122.**

**53. *Shakya VC. Simultaneous laparoscopic management of Morgagni hernia and cholelithiasis: two case reports. BMC Res Notes. 2015 Jul 1;8:283. doi: 10.1186/s13104-015-1249-y. PMID: 26126719; PMCID: PMC4487111.***

**54. *Soldo I, Baća I, Sever M, Zoričić I, Grbavac M, Troskot Perić R, Čala Z, Karaman Ilić M, Soldo A, Bakula B. Laparoscopic Treatment of Morgagni Hernia: Report of Three Cases. Acta Clin Croat. 2017 Jun;56(2):318-322. doi: 10.20471/acc.2017.56.02.16. PMID: 2.* PMID: 29485800.**

**55. *Ryan JM, Rogers AC, Hannan EJ, Mastrosimone A, Arumugasamy M. Technical description of laparoscopic Morgagni hernia repair with primary closure and onlay composite mesh placement. Hernia. 2018 Aug;22(4):697-705.* doi: 10.1007/s10029-018-1760-x. Epub 2018 Mar 19. PMID: 29556855.**

**56. Kumar A, Karn R, Khanal B, Sah SP, Gupta R. Laparoscopic approach for diaphragmatic hernia repair in adult: our experience of four cases. J Surg Case Rep. 2020 Jun 19;2020(6):rjaa178. doi: 10.1093/jscr/rjaa178. PMID: 32595927; PMCID: PMC7303022.**

**57. *Durak E, Gur S, Cokmez A, Atahan K, Zahtz E, Tarcan E. Laparoscopic repair of Morgagni hernia. Hernia. 2007 Jun;11(3):265-70. doi: 10.1007/s10029-006-0178-z. Epub 2006 Dec 20. PMID: 17180632.***

**58. Leeflang E, Madden J, Ibele A, Glasgow R, Morrow E. Laparoscopic management of symptomatic congenital diaphragmatic hernia of Morgagni in the adult. Surg Endosc. 2021 Jan 12. doi: 10.1007/s00464-020-08259-z. Epub ahead of print. PMID: 33438077.**

**59. *Yavuz N, Yiğitbasi R, Sunamak O, As A, Oral C, Erguney S. Laparoscopic repair of Morgagni hernia. Surg Laparosc Endosc Percutan Tech. 2006 Jun;16(3):173-6. doi: 10.1097/00129689-200606000-00013. Erratum in: Surg Laparosc Endosc Percutan Tech.* 2006 Aug;16(4):table of contents. PMID: 16804464.**

**60. Young MC, Saddoughi SA, Aho JM, Harmsen WS, Allen MS, Blackmon SH, Cassivi SD, Nichols FC, Shen KR, Wigle DA. Comparison of Laparoscopic Versus Open Surgical Management of Morgagni Hernia. Ann Thorac Surg. 2019 Jan;107(1):257-261. doi: 10.1016/j.athoracsur.2018.08.021. Epub 2018 Oct 6. PMID: 30296422.**

**61. Oppelt PU, Askevold I, Bender F, Liese J, Padberg W, Hecker A, Reichert M. Morgagni-Larrey diaphragmatic hernia repair in adult patients: a retrospective single-center experience. Hernia. 2020 Feb 29. doi: 10.1007/s10029-020-02147-0. Epub ahead of print. PMID: 32112200.**

**62. *Arikan S, Dogan MB, Kocakusak A, Ersoz F, Sari S, Duzkoylu Y, Nayci AE, Ozoran E, Tozan E, Dubus T. Morgagni's Hernia: Analysis of 21 Patients with Our Clinical Experience in Diagnosis and Treatment. Indian J Surg. 2018 Jun;80(3):239-244.* doi: 10.1007/s12262-016-1580-0. Epub 2017 Jan 6. PMID: 29973754; PMCID: PMC6014954.**

**63. *Kao AM, Ayuso SA, Huntington CR, Sherrill WC, Cetrulo LN, Colavita PD, Heniford BT. Technique and Outcomes in Laparoscopic Repair of Morgagni Hernia in Adults.* J Laparoendosc Adv Surg Tech A. 2021 Jul;31(7):814-819. doi: 10.1089/lap.2021.0038. Epub 2021 May 12. PMID: 33979533.**

**64. *Onafowokan OO, Khosa K, Bonatti H. Laparoscopic Repair of Morgagni Hernia Combined with Right Hemicolectomy for Bleeding Ascending Colon Carcinoma Lodged within the Chest: A Case Report and Review of the Literature. Case Rep Surg.* 2021 Jul 19;2021:5533203. doi: 10.1155/2021/5533203. PMID: 34336347; PMCID: PMC8315875.**

**65. *Pazouki A, Mohammadyari F, Maleknia SA, Meymand FE, Gholizadeh B. Laparoscopic repair of an incarcerated Morgagni Hernia in a COVID-19-positive patient: .* A video case report. Int J Surg Case Rep. 2022 Apr;93:106846. doi: 10.1016/j.ijscr.2022.106846. Epub 2022 Feb 24. PMID: 35306334; PMCID: PMC8941208.**

**66. *Altın Ö, Kaya S, Sarı R. Laparoscopic and single incision laparoscopic repair of Morgagni hernia in adults. Turk Gogus Kalp Damar Cerrahisi Derg.* 2021 Oct 20;29(4):513-519. doi: 10.5606/tgkdc.dergisi.2021.20983. PMID: 35096449; PMCID: PMC8762901.**

**67. *Taskin M, Zengin K, Unal E, Eren D, Korman U. Laparoscopic repair of congenital diaphragmatic hernias. Surg Endosc. 2002 May;16(5):869. doi: 10.1007/s004640042025. Epub 2002 Feb 28. PMID: 11997842.***
